# The International Mouse Strain Resource (IMSR): cataloging worldwide mouse and ES cell line resources

**DOI:** 10.1007/s00335-015-9600-0

**Published:** 2015-09-15

**Authors:** Janan T. Eppig, Howie Motenko, Joel E. Richardson, Beverly Richards-Smith, Cynthia L. Smith

**Affiliations:** Mouse Genome Informatics, The Jackson Laboratory, Bar Harbor, ME 04609 USA

## Abstract

The availability of and access to quality genetically defined, health-status known mouse resources is critical for biomedical research. By ensuring that mice used in research experiments are biologically, genetically, and health-status equivalent, we enable knowledge transfer, hypothesis building based on multiple data streams, and experimental reproducibility based on common mouse resources (reagents). Major repositories for mouse resources have developed over time and each has significant unique resources to offer. Here we (a) describe The International Mouse Strain Resource that offers users a combined catalog of worldwide mouse resources (live, cryopreserved, embryonic stem cells), with direct access to repository sites holding resources of interest and (b) discuss the commitment to nomenclature standards among resources that remain a challenge in unifying mouse resource catalogs.

## Introduction

The IMSR began as a collaboration between MRC Harwell and the Mouse Genome Database (MGD) at the Jackson Laboratory to provide the research community with a common site for finding mouse resources (Eppig and Strivens 1999). The IMSR has since grown to be a multi-institutional international collaboration supporting the research community that uses mouse as a model system for studying biology and disease (Eppig et al. 2005). The goal of the IMSR remains to provide a web searchable catalog to assist investigators in finding the mouse resources needed for their studies. The IMSR produces a continuously updated global resource for scientific investigators. Collaborating repositories contribute data on the resources held in their individual repositories via FTP files regularly submitted to the IMSR database. These data are processed and integrated with data from other repositories, creating a collective global compendium of resources available worldwide. These integrated resources are then provided on a searchable website (www.findmice.org) with links to each repository, the repository’s ordering form, the repository’s web page describing each holding, and links to the Mouse Genome Database (MGD, www.informatics.jax.org) for information about the gene(s) involved, the specific mutations and their genetic backgrounds, the abnormal phenotypes manifested by the mice, and human disease model assertions based on author-reported experimental data with links to the relevant MGD disease page and to OMIM (Online Mendelian Inheritance in Man) records for human disease descriptions.

The IMSR is a dynamic data system. As repositories submit their current holdings, IMSR performs quality control (QC) checks on the data and, if the data files are properly formed, processes the files and refreshes the web presentation to reflect current repository holdings. Ongoing curation provided by MGD staff feeds back to the repositories to continually improve the standardization of nomenclature among repositories and thus iteratively improving the ability of researchers to effectively query IMSR (and their representative repository sites) using standard official nomenclature and retrieve complete results.

## International mouse strain resource (IMSR): integrating repository holdings worldwide

### Principles and design

The goal of the IMSR is to provide an online searchable web-based catalog of mouse resources available globally, including inbred, mutant, and genetically engineered mice, cryopreserved embryos and gametes, and ES cell lines. The IMSR website provides, for each strain or cell line, links for ordering, links to the repositories’ strain description, and links to phenotype and disease model data. Mouse repositories of any size and in any location are welcome to contribute data about their mouse resource holdings, providing those holdings are available to investigators who request resource access. This does not mean the resources are without cost, but that they are available to researchers. Most repositories charge customers to recover their cost of operating, and maintaining and shipping mouse resources. In addition, IMSR expects that resources will update their holdings on a regular basis. Many active repositories provide new data files on a weekly basis. Individual investigators are welcome to contribute to IMSR as a small repository as well, if they are distributing their resources and will ship their unique mouse mutants without special restrictions.

### Repositories contributing to IMSR

There are currently 20 repositories and repository consortia (representing 46 individual repository sites) listing mouse resource holdings in IMSR (Table 1). These collectively hold 32,396 mouse strains (as live stocks, cryopreserved embryos, and cryopreserved gametes) and 209,328 mutant ES cell lines as of May 15, 2015 (Table 1). Of these, approximately 1300 strains exist as both ES cell lines and as some animated form (largely as cryopreserved sperm or embryos). There is virtually no duplication in strain holdings between repositories. However, at any given time, a repository may have available multiple forms of a given strain (e.g., frozen embryos or live mice) either through the dynamic cycle of cryopreservation, recovery, breeding, and re-cryopreservation that happens in providing or restoring a given repository’s strain holdings or as a matter of repository policy to store strains in multiple states (e.g., as cryopreserved embryos and sperm).

**Table 1:** IMSR (www.findmice.org) repositories’ holdings^a^ (data as of May 15, 2015)

Repository/consortium	Abbreviation	Region	Strains^e^	ES cell lines
Australian Phenome Bank^b^	APB	Australia	170	0
Center for Animal Resources and Development	CARD	Japan	1292	31
Canadian Mouse Mutant Repository	CMMR	Canada	167	13,653
European Mouse Mutant Archive^c^	EMMA	Europe	4345	0
Dr. Elizabeth M. Simpson, Ph.D.	EMS	Canada	4	0
MRC Harwell (HAR)	HAR	U.K.	2326	0
JAX Mice	JAX	U.S.A.	8146	22
Knock-out Mouse Project (KOMP)	KOMP	U.S.A.	1526	13,362
Mutant Mouse Regional Resource Centers^d^	MMRRC	U.S.A.	4574	28,449
MUGEN Mouse Database	MUGEN	Greece	75	0
National Cancer Institute at Frederick	NCIMR	U.S.A.	132	0
National Institute of Genetics	NIG	Japan	142	0
National Resource Center for Mutant Mice	NRCMM	China	144	1
Oriental BioService, Inc.	OBS	Japan	29	0
Oak Ridge Collection at JAX	ORNL	U.S.A.	910	0
RIKEN BioResource Center	RBRC	Japan	4042	1747
National Applied Research Laboratories	RNRC-NLAC	Taiwan	236	0
Taconic Biosciences	TAC	U.S.A.	3845	0
Texas A&M Institute for Genomic Medicine	TIGM	U.S.A.	180	142,538
Wellcome Trust Sanger Institute	WTSI	U.K.	102	9525
TOTAL			32,396	209,328

^a^An additional 5 repositories have registered with IMSR, but have not yet submitted data

^b^The Australian Phenome Bank is an Australian consortium including repositories at the Australian National University; Monash University; University of Melbourne; Walter and Eliza Hall Institute of Medical Research; the Peter MacCallum Cancer Centre; Macquarie University; South Australian Health and Medical Research Institute; the Animal Resource Centre; and the Harry Perkins Institute of Medical Research

^c^The European Mouse Mutant Archive is a consortium including repositories at CNR Instituto di Biologia Cellulare, Monterotondo, Italy; CNRS Centre de Distribution de Typage et d’Archivage Animale, Orleans, France; MRC Mammalian Genetics Unit, Harwell, UK; Karolinska Institute, Stockholm, Sweden; Helmholtz Zentrum München, Munich, Germany; Wellcome Trust Sanger Institute, Hinxton, UK; Institut Clinique de la Souris, Strasbourg, France; CNB-CSIC, Centro Nacional de Biotecnologia, Madrid, Spain (Wilkinson et al. 2010)

^d^The Mutant Mouse Regional Resource Centers is a consortium of repositories in the U.S.A. with facilities at the MMRRC-Jackson Laboratory; University of California, Davis; University of Missouri; and University of North Carolina, Chapel Hill

^e^Strain number is unique holdings, including live mouse colonies, as well as cryopreserved embryos, ovaries, and sperm

Large-scale mutagenesis and analysis projects including the International Mouse Phenotyping Consortium (IMPC) mice recovered from ES cell line knockout mutations and mice generated in subsequent crosses to cre-deleter mice for removal of neo-cassettes (Brown and Moore 2012; Mallon et al. 2012) and several *N*-ethyl-*N*-nitrosourea (ENU) targeted phenotype screens (Li et al. 2015; Arnold et al. 2012; Goldowitz et al. 2004; Nolan et al. 2000; Hrabé de Angelis et al. 2000; Justice et al. 1999) have or are generating a significant number of new genetically defined mice that are actively being archived in existing mouse repositories. In addition, the adoption of gene-editing technologies using TALENs (zinc-finger nucleases, transcription activator-like effector nucleases) and CRISPR/Cas9 (clustered regularly interspaced short palindromic repeats/CRISPR associated system) (Aida et al. 2015; Singh et al. 2015; Sung et al. 2012) by the IMPC and the larger mouse genomics community will contribute to additional expansions to repository inventories.

### Informatics infrastructure

**Fig. 1:**
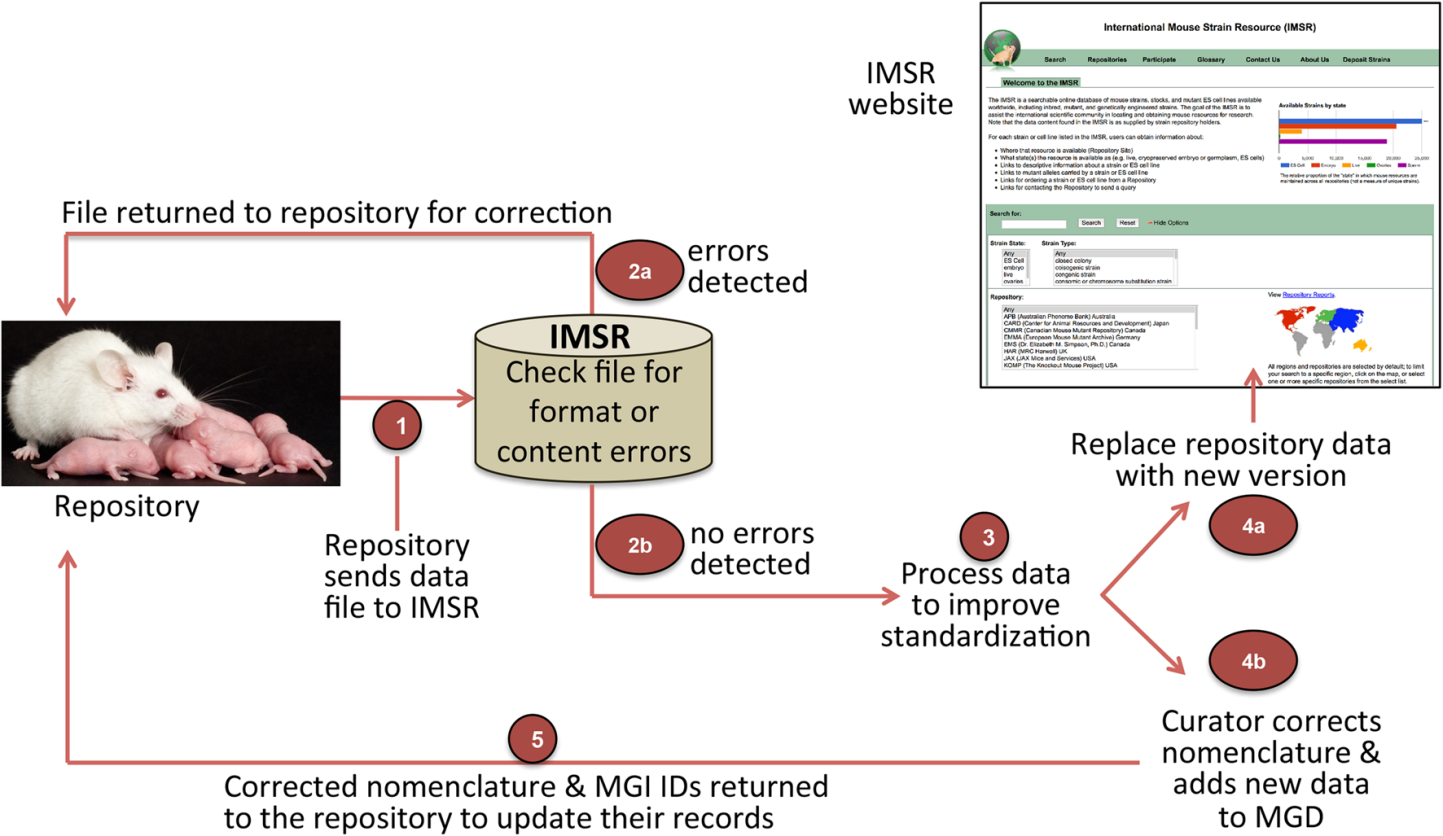
Overview of IMSR process. (1) Individual repository databases initiate data contribution to IMSR by providing a format-specified text file to IMSR via a private FTP site. Automated checks confirm that the incoming data file is properly formatted and that fields contain the data of correct type. If not, (2a) data are returned with comment to inform the repository of error corrections required for submission. If data pass this automated check, (2b) data are processed to compare them to MGD data (3) standardizing nomenclature to allow better web links and producing nomenclature and new data reports for curator action. Repository data are replaced with a new version (4a) for display on the IMSR website and (4b) curators correct and/or update repository submitted nomenclature and add new data to MGD. The curation results also are returned to the repository (5) for updating their files with the correct nomenclature for the alleles, genes, and strains that they hold in their resource

An overview of the IMSR system is shown in Fig. 1. Data providers deposit files in a defined format on a private FTP site. The format is a simple tab delimited text file whose fields are specified on an IMSR help page (http://www.findmice.org/participate). An automated process checks the deposit area hourly. New submissions are scanned for formatting and content errors (e.g., missing values or IDs that do not designate valid genes). Any errors are communicated to the providers via email along with instructions for needed fixes and contact information for obtaining assistance. Submissions that pass the error checking process are archived, then processed to improve content against MGD data, indexed by Lucene (http://lucene.apache.org), and made available via our Solr instance (http://lucene.apache.org/solr/). IMSR is a noSQL system; all queries and page generation are supported by Solr/Lucene, which provides very fast response times. IMSR currently uses Solr 5.2 running in a WildFly 8.2 container. A parallel system is used for development and testing.

Concurrently, after a newly submitted data file passes automated checks, comparison with MGD data will reveal any data inconsistencies (e.g., incorrect strain names, mismatched IDs, or gene or allele nomenclature in need of updates). These will be curated and corrections returned to the repository provider so they can update their records. In this manner, repositories become more nomenclature accurate/current and iteratively improve their data; and users benefit from future loads of corrected data being more readily searchable using standard allele, gene, and strain designations.

### User interface: the IMSR website

**Fig. 2:**
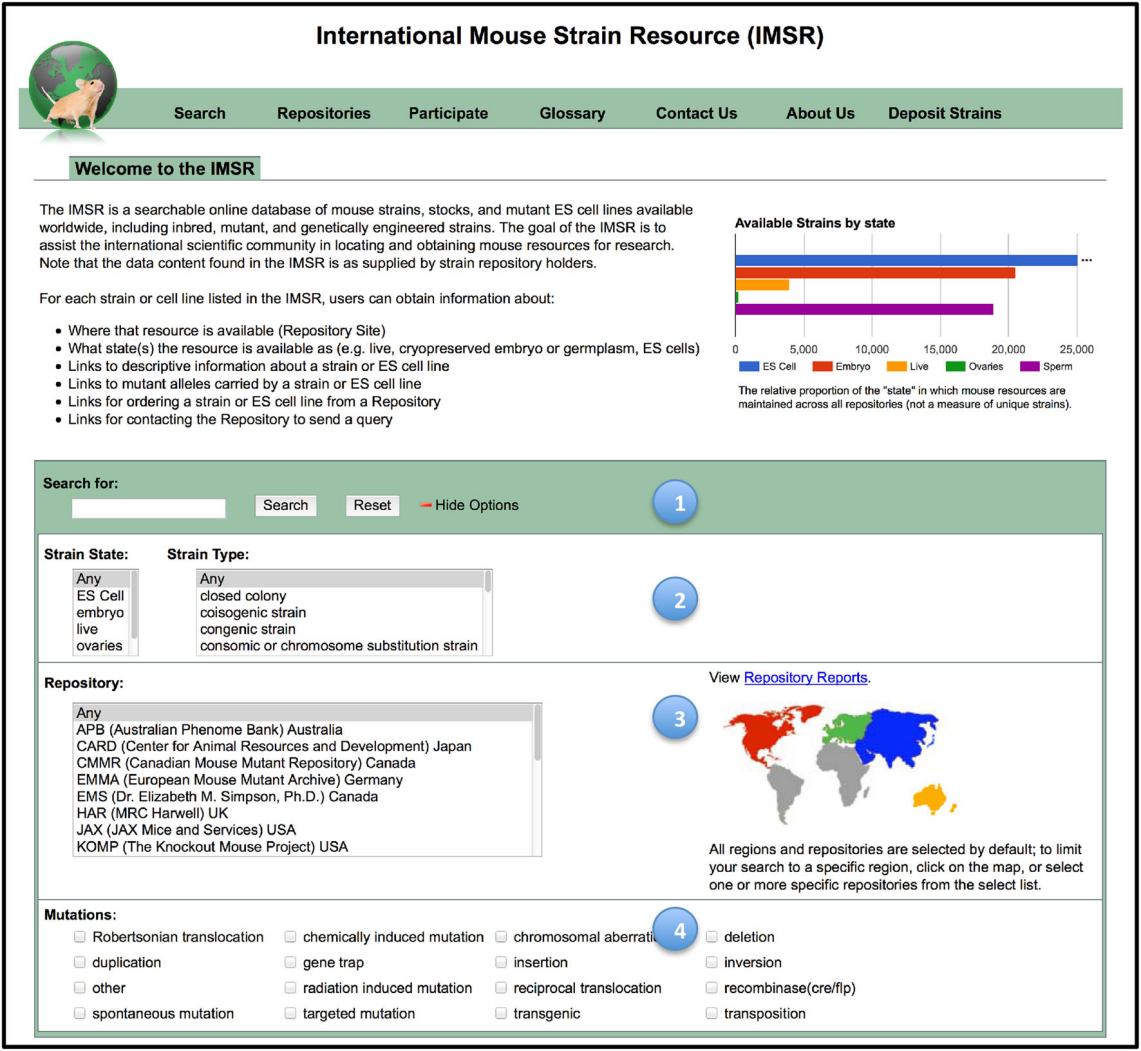
IMSR homepage/search page. (www.findmice.org). This web page introduces IMSR and is the search form for IMSR content. The search section has (1) a *Quick Search* option (*green bar* located mid-page), where one may type in a single field a mutant allele, gene, or strain and click the search button; (2) a strain search section where one may further specify the “state” (e.g., embryo, live) in which the resource is maintained and the “strain type” which defines how the strain is bred; (3) a Repository search limiting results to one or more specific repositories of choice, or using the map, limit one’s search to a particular continent; and (4) mutation type, specifying how the mutant allele of interest was created (e.g., gene trap, transgenic, targeted)

**Fig. 3:**
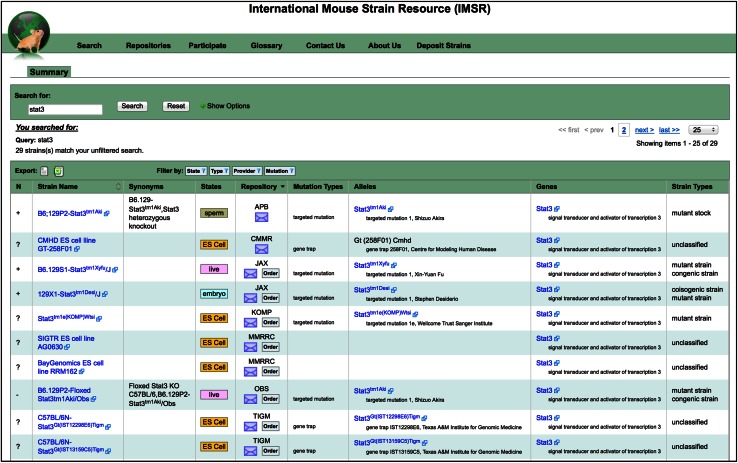
Example IMSR search result. This screenshot shows the first 10 (of 29) lines returned when searching IMSR for strains with mutations in the *Stat3* (signal transducer and activator of transcription 3) gene. *Columns* shown include (1) “*N*” for nomenclature, where + is correct; − is incorrect, and ? is unreviewed; (2) *Strain Name*, each strain name links to the strain description page on the respective repository’s website; (3) *Synonyms*, other names that this strain has been called, including former names and lab-jargon names; (4) *States* are how the mouse resource exists—live, cryopreserved embryo, sperm, or ova, or ES cell line; (5) the *Repository* *column* provides two links, when available—an email link to user assistance at the repository site and a link to “order” the mouse resource from the repository site; (6) *Mutation type*, as provided by repository data; (7) *Alleles*, symbol and name, as provided by repository or enhanced by MGD linking; (8) *Genes* indicating those mutated in the mouse stock in question; and (7) *Strain Type* defined by breeding scheme, if known. Buttons to export the current results are found above the strain name column

Users access IMSR primarily via a web-based interface (www.findmice.org). Searches can be performed using one or many parameters, including strain parameters, genetic parameters, and repository name/location. Strain parameters include the strain/stock designation, the strain ID, the state in which the strain/resource is maintained (live, cryopreserved embryo, cryopreserved ovary, cryopreserved or freeze-dried sperm, or ES cell line), and the strain type. Genetic parameters include the symbol or name of the phenotypic allele or gene of interest carried in the strain, the relevant allele or gene accession ID, and the type (origin) of the mutation and its chromosomal location. Repository parameters include the name of one or more specific repositories, or the selection of all repositories in a geographical regional location (Fig. 2). The results of a search are returned in tabular format, with each row in the table representing one unique genetic strain from a given repository. [Note, therefore, that if a repository holds a strain in multiple states, the strain is only listed once; but each “state” status is provided]. Search results can be exported in text or Excel format. Figure 3 shows 10 rows of the 29 rows returned when searching for strains carrying mutations in the *Stat3* gene (as of May 15, 2015).

**Fig. 4:**
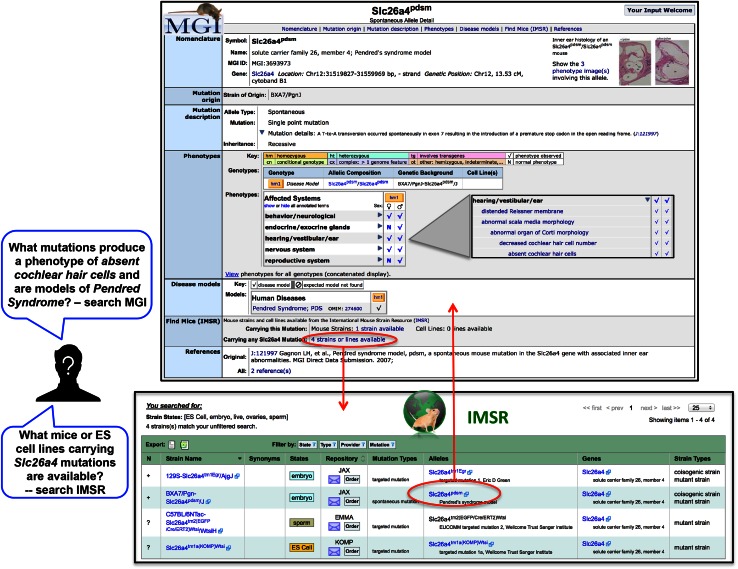
Interplay between MGD and IMSR that maximizes user access. A user searching for a particular strain or mutations in a particular gene can quickly find these resources using the IMSR interface (see Figs. 2, 3 and text). However, if a user does not know the strain or mutant desired, but wants to search for mouse resources on the basis of phenotype or disease model, he/she would start with a phenotype/disease search of MGD. Using either entry point, the user can easily follow links between them. For example, a user who searched MGD for a phenotype of *absent cochlear hair cells* or the human disease *Pendred Syndrome* would view phenotype detail pages such as the one shown for *Slc26a4* ^*pdsm*^ (*top panel*). From this page, a link to IMSR (*red circle*) will lead the user to IMSR for a list of strains and cell lines available with *Slc26a4* mutations (*bottom panel*). The link shown from IMSR (*red circle*) will lead to the relevant detailed phenotype/disease page in MGD

But how does an investigator who does not know what strain or what mutant he/she may need approach the IMSR? The key is found in the reciprocal links and complementary information contained in MGD (phenotype and disease model data) and the IMSR (strain listings). Figure 4 illustrates the interplay between these sites conceptually. A user directs questions that are phenotype or disease model oriented in nature to the MGD database where he/she can then view the specifics of a mutant phenotype or learn what human disease(s) this mutant is used to model. Each such page in MGD detailing the phenotype and disease models for a given mutant links directly to IMSR for users to physically locate strains or ES cell line resources containing the mutant in question. Similarly, a user of IMSR, when viewing a set of strains and ES cell lines that carry mutations in a particular gene can, for any of those strains, link directly to the MGD detail page describing the phenotype and disease models.

### Data standards and challenges in maintaining and updating IMSR

The largest challenge of maintaining IMSR is the variability in data quality and completeness among the data files submitted to IMSR from different repositories. Although there is documentation about data fields and required format, data received, particularly from smaller repositories with less sophisticated informatics infrastructure, may be incomplete, or the repositories may not have some critical information from the original source that generated the mice. However, for any given strain holding submitted in the repository file, if the minimum data fields are provided (strain ID, strain name, state, strain type), that strain can display in the IMSR website, but only limited links to other information resources will be possible.

A second challenge for IMSR is the use of non-standard nomenclature in the gene, mutant allele, and strain designations provided to IMSR. In processing incoming files, IMSR scripts are run that attempt some automatic data field completion (e.g., if the repository provided a nomenclature-correct mutant allele, but left the gene field blank, the correct gene can be inferred). Other scripts compare incoming data with MGD data to allow withdrawn nomenclature or nomenclature synonyms to be replaced with the correct symbols and names on the IMSR website. These automatic corrections allow links to gene and allele data that would otherwise not be possible.

Many nomenclature errors cannot be easily interpreted as described above. These are displayed on the IMSR website ‘as is.’ A curator reviews logs of data errors from incoming repository files and returns corrections to the repository, where possible. It remains incumbent upon the repository to update their own holdings’ database with corrected nomenclature and IDs. This feedback is intended to improve the repository’s own site, as well as to ensure that the next data file provided to IMSR is correct and does not again appear in the error log.

## Perspective on mouse repositories

Mouse resources are important for all who use mouse as an experimental system. The ability to obtain genetically defined mouse resources of known health status is key for producing results that are reproducible and can be built upon in future work. Mouse repositories focus their efforts on providing the highest quality, genetically tested, and strain background-defined resources possible. The IMSR facilitates their work by assisting investigators worldwide with access to those resources, wherever they exist. The last decade has seen an explosion of new mouse resources produced aided by new genetic technologies. These are making their way into repositories, with a rapid uptake by experimental scientists. The work of the global repository network and the IMSR remain important pieces of the infrastructure fabric for biological and disease model research of the future.
